# Recognition and Evaluation of Clinical Section Headings in Clinical Documents Using Token-Based Formulation with Conditional Random Fields

**DOI:** 10.1155/2015/873012

**Published:** 2015-08-26

**Authors:** Hong-Jie Dai, Shabbir Syed-Abdul, Chih-Wei Chen, Chieh-Chen Wu

**Affiliations:** ^1^Department of Computer Science and Information Engineering, National Taitung University, Taitung 95092, Taiwan; ^2^Graduate Institute of Biomedical Informatics, College of Medical Science and Technology, Taipei Medical University, Taipei 11042, Taiwan

## Abstract

Electronic health record (EHR) is a digital data format that collects electronic health information about an individual patient or population. To enhance the meaningful use of EHRs, information extraction techniques have been developed to recognize clinical concepts mentioned in EHRs. Nevertheless, the clinical judgment of an EHR cannot be known solely based on the recognized concepts without considering its contextual information. In order to improve the readability and accessibility of EHRs, this work developed a section heading recognition system for clinical documents. In contrast to formulating the section heading recognition task as a sentence classification problem, this work proposed a token-based formulation with the conditional random field (CRF) model. A standard section heading recognition corpus was compiled by annotators with clinical experience to evaluate the performance and compare it with sentence classification and dictionary-based approaches. The results of the experiments showed that the proposed method achieved a satisfactory *F*-score of 0.942, which outperformed the sentence-based approach and the best dictionary-based system by 0.087 and 0.096, respectively. One important advantage of our formulation over the sentence-based approach is that it presented an integrated solution without the need to develop additional heuristics rules for isolating the headings from the surrounding section contents.

## 1. Introduction

Electronic health record (EHR) is a digital data format that collects electronic health information about an individual patient or population. The use of EHRs offers advantages not only for direct patient care, but also for secondary purposes such as clinical research, quality improvement, and public health. For example, the use of EHRs eliminates the manual task of extracting data from charts and also promotes the access, retrieval, and sharing of clinical information. Sophisticated data mining techniques can then be applied to EHR data to understand the trends and differences between various patient populations. A large collection of EHRs is also invaluable to the development of computer-aided diagnostic tools [1]. However, according to a study by Capurro [2], approximately 50% of EHR data collected from sources like clinical notes, radiology reports, and discharge summaries is stored as free text. Unstructured format as such makes it difficult to retrieve meaningful information from EHRs. In light of this issue, information extraction (IE) techniques have been applied to unstructured parts of EHRs to assist clinical decision support and foster analysis and clinical research [3].

Recognition of clinical entities such as drugs and diseases in clinical narratives is one of the fundamental tasks of mining information from EHRs. Several clinical IE systems, such as MedLEE [4], MetaMap [5], and cTAKES [6], have been developed to support clinical entity recognition tasks. However, the judgment of clinical data cannot be done solely at the named entity level. For instance, “coronary heart disease” has different types of clinical significance in sections such as past medical history and family medical history. The frequent use of author- and domain-specific idiosyncrasies, acronyms, and abbreviations within different parts of an EHR also increases the difficulty for IE systems to correctly interpret the categories of named entities. For example, the acronym “BS” means “blood sugar” in the laboratory section but indicates “bowel sounds” in the section of abdominal exams [7]. The development of clinically structured entry systems seems to be the optimal solution, which can preformat information into predefined section fields. Unfortunately, it is inflexible and has been revealed to significantly interfere with the clinical workflow and slow users down [8]. Therefore, an unstructured documentation method is still used by most healthcare providers in order to reserve clarity and flexibility.

With flexible documentation, text processing algorithms such as recognition of section headings can be used to produce structured data once clinical documentation is complete.  Table 1 displays a sample of discharge summary, which is transcribed to provide an overview of a patient's hospitalization from admission to discharge. Evidently, a discharge summary can cover listed items including the admission/discharge dates, diagnoses, discharge physical examinations, key laboratory data, medications that the patient is on at the time of discharge, narratives about the patient, the circumstances leading up to admission, and the patient's progress and treatments from admission to discharge. Recognizing these section headings can not only improve the quality of IE but also provide an enhanced experience in reading and accessing EHRs. Due to the versatility of a patient's condition and treatment, the layout format including the employed section headings may vary, thus increasing the complexity of section-heading recognition.

The challenges in recognizing section headings in EHRs can be summarized as follows. First of all, the names of section headings do not follow a universal system. For the section of a chief problem, possible names may include “chief complaint”, “presenting complaint(s)”, “presenting problem(s)”, “reason for encounter”, or even the use of the abbreviation “CC” as shown in Table 1. Occasionally, the same section name may infer different definitions. “CC” can refer to “chief complaint” in a discharge summary or “carbon copy” in a clinical narrative written in an email. The use of capitalization and colons for section names can be very inconsistent within and across documents. Furthermore, the hierarchies of sections vary from record to record. For instance, “Laboratory” and “Radiology” may be two independent sections, or both may be placed together under the “Data” section. “Impression and assessment” may be separated individually, or they may be merged together into one section. “Impression” section can contain the overall diagnosis of a patient, or it can be a subsection of image studies. Therefore, section-heading recognition approaches entirely based on dictionaries or patterns are not always competent.

In view of this issue, this paper compiled a section-heading recognition corpus on top of the dataset released by the i2b2 2014 shared task [9] and presents a machine learning approach based on the conditional random fields (CRF) model [10] to handle the section-heading recognition task for EHRs. Based on the assumption that the narratives following a recognized section heading should belong to this corresponding section, this work modeled the task as a sequential token labeling problem in a given text, which differs from most of the previous works [11, 12] that formulated the problem as a sentence-by-sentence classification task. The compiled corpus along with the developed model and section-heading recognition tool is publicly available at https://www.sites.google.com/site/hongjiedai/projects/nttmuclinicalnet and http://btm.tmu.edu.tw/nttmuclinicalnet/ in an attempt to facilitate clinical research.

## 2. Materials and Methods

### 2.1. Section-Heading Recognition Corpus

To the best of our knowledge, currently there is no openly available corpus annotated with medical section-heading information. Therefore, this work compiled a corpus for developing and evaluating the proposed method. The dataset of track 2 of the i2b2 2014 shared task was selected, which contains 1304 medical documents of different document types including discharge summaries, procedural notes, and emails between the primary physician and the consultant. Most documents are related to patients with coronary artery disease and/or diabetes. We followed our previous work [13] to employ the section-heading strings listed in the clinical note section header terminology (SecTag) [7] to tag all plausible candidate heading mentions. In contrast to the previous work in which only one domain expert annotated the dataset, the machine-generated annotations in this work were loaded into the brat annotation tool [14] and then manually corrected by the second, third, and fourth authors of this paper. The first annotator is a clinical expert who is a medical doctor with linguistic annotation training. The second annotator is an experienced medical doctor with clinical working experience in internal medicine for more than 15 years. The third annotator is an experienced medical doctor and a professor in medical informatics.

**Figure 1:**
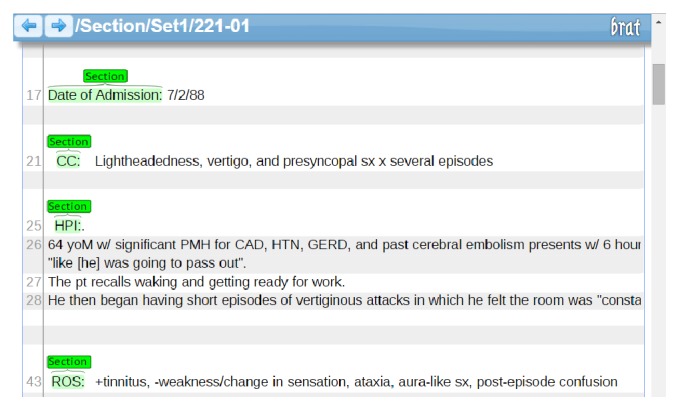
An annotated document sample on brat.

For the manual annotation task, the annotators were instructed to only annotate the topmost section headings. We searched literatures extensively for standard section-heading definitions for discharge summaries but observed that each country adopted different definitions. Hence, this work followed the discharge summary exchange standard (http://emrstd.mohw.gov.tw/strdoc/default.aspx) defined by the electronic medical record exchange centre built by the Ministry of Health and Welfare of Taiwan to define the topmost sections. Based on this standard, section headings that can be viewed as subsections themselves or are followed by contents belonging to a superior section were removed from the annotations, regardless of their section level in other documents. For instance, if both the “Laboratory” and “Radiology” sections existed in EHR but can be considered as subsections of the “Data” section, then only the superior section “Data” was annotated. On the contrary, if “Laboratory” and “Radiology” were two separate sections without a common superior section, then both sections were annotated. Consider another example in which “Impression” was annotated if it was the topmost section. However, if the content of the “Impression” section clearly contained the data of certain reports, such as X-ray or echography, and trailed behind other section headings like “Cardiac Echography” or “Chest X-ray”, then the annotation of the “Impression” section was removed. Furthermore, if the name of a topmost section consisted of two merged concepts, it was still annotated as one section heading. For instance, some documents combined the sections “Impression” and “Plan” as one section “Impression/Plan”, while others recorded both sections independently. Finally, section headings were further extended to include punctuation marks and parentheses, such as “Chief Complaints:” and “Medications (**updated 8/28/70**)”.  Figure 1 shows an example of the annotated document within the brat annotation tool.

The interannotator agreement probability was 0.934. The main source of disagreement was that the annotators sometimes overlooked a few headers in a given note. Annotators also had different opinions on some unusual or idiosyncratic section headings, such as “carbon copy”. In addition, one annotator labelled the content of emails that was used to describe medical activities, such as “medical regimen” and “on exam”. However, these terms should not be annotated as section headings. The final corpus contained 13,962 section headings in 1304 documents and 1335 unique section headings. On average, there were 10.7 sections per document. The distribution of section headings was manually analysed by one of the annotators and is presented in Table 2.

Among all annotated sections, 5.7% comprised the “Chief Complaints” section, which was found to be presented in several alternative spellings such as CC, chief, and reason. 6.0% of the sections were the “Present Illness” section. “Personal Histories” made up 19% of the sections, which included subsections like social history, medication, allergy, substance, marital status, and activity and general health status. 3.4% comprised the “Family Histories” section, while 7.9% comprised the “Physical Examinations” section. In addition, 2.8% consisted of the “Laboratory Examinations” section, and less than 1.0% of the annotated sections were the “Radiology Reports”. Less than 1.0% was the “Data” sections, which included the laboratory and radiology results. Finally, 6.3% of the sections belonged to “diagnosis” or “impressions”, 3.3% were “Plans” or “Recommendations”, and the remaining 43.6% contained other section names such as patient name, physician name, hospital name, identity number, and carbon copy and merged sections such as “assessment and plan”.

Moreover, the annotated section-heading occurrences in all EHRs have been analysed to examine the distribution of explicit and implicit section-heading boundaries. The results revealed that 44.32% of the section headings appeared in one single line without any other content, while 55.58% of the section headings are accompanied by other information. The latter includes mixtures of different content and section headings on the same line. For instance, the section headings “Habits” and “Review of Systems” are surrounded by additional content in the line in the phrase “… prn.#180 Tablet(s)**Habits** Tobacco: no smoking**Review of Systems** No change in wt. No …”. This analysis exposed the potential problem of the sentence-by-sentence classification formulation. Without further postprocessing of the classified results, the sentence-based classification formulation cannot recognize the section headings and their corresponding content when they coexist on the same line.

### 2.2. Formulation of the Section-Heading Recognition Problem

Previous studies formulated the section-heading recognition problem as a sentence classification task [11]. By contrast, this work formulated the section-heading recognition problem as a token-based sequential labeling task and employed the IOB tag scheme to represent annotations of section headings. The B tag indicates that the current token is the beginning of a section-heading boundary, the I tag indicates tokens inside the boundary of a section heading, and the O tag represents the tokens outside a section heading. For example, after applying the IOB tag scheme, the paragraph “Date of Admission: 7/2/88” in Table 1 was annotated as “Date_**/B**_ of_**/I**_ Admission_**/I**_:_**/I**_ 7_**/O**_/_**/O**_2_**/O**_/_**/O**_8_**/O**_8_**/O**_”, in which the assigned tag was subscripted and highlighted in bold.

Based on this formulation, the narratives between the preceding and subsequent recognized section headings should be annotated with the O tag and considered as the body of the leading section. Using Table 1 as an example, the narratives “64 yoM w/significant PMH for CAD,…Thept recalls…” between the two sections HPI (history of present illness) and ROS (review of system) were considered as the body of the HPI section.

### 2.3. Preprocessing and Model Features

For a given EHR, the following preprocessing was applied. First, the raw text was extracted from the clinical data while retaining the original line breaks. The text distinguished by the line breaks was then processed by the MedPost tagger [15] to further split it into lines of texts that consist of tokens. Each line was then aligned with the annotations of domain experts to generate training instances for the machine learning model.

The machine learning model utilized herein is the CRF model, which is an undirected graphical model that is trained to maximize a conditional probability of random variables. In this study, the linear-chain CRF model was employed to recognize sequentially the boundaries of section headings for a given tokenized textual sequence (CRF++, which is available at http://taku910.github.io/crfpp/, was used to implement the linear-chain CRF model). Given an input sequence of tokens *W*, a linear-chain CRF model computes the probability associated with its corresponding hidden labelled sequence *Y* as

(1) pλY ∣ W=1ZWexp⁡∑c∈C ∑iλifiyc−1,yc,W,c,

where *Z*(*W*) is the normalization factor that makes the probability of all state sequences sum up to one, *C* is the set of all cliques in this textual sequence, and *c* is a single clique that reflects the position of the current token. The function *f*_*i*_ (*y*_*c*−1_, *y*_*c*_, *W*, *c*) is a binary-valued feature function whose weight is *λ*_*i*_. Large positive values of *λ*_*i*_ indicate a preference for such corresponding feature. For each token, a set of feature functions was defined and their feature values were extracted and trained with the CRF model to build the section-heading recognizer. The following subsections elaborate the features developed for this work.

### 2.4. Word Features

Evidently, the word of a target token and words preceding or following the target token can be useful in determining the target token's assigned tag. This work used the content window size of five to extract word features, including the two preceding words, the current word, and the two following words. In addition, the advantages of normalizing words when encoding them as features were shown in many information extraction tasks formulated as sequential labelling [16]. Therefore, this work normalized all words within a context window by transforming all words into lower case and encoding all numeric values as the value 1.

### 2.5. Affix Features

An affix refers to a morpheme that is attached to a base morpheme to form a word. This work employed two types of affixes: prefixes and suffixes. Some prefixes and suffixes can provide good clues for classifying section headings. For example, words which end in “Hx” are related to medical historical information, as PSurHx refers to the “past surgical history” section. This work used the length of 2 characters for prefixes and suffixes.

### 2.6. Orthographic Features

Although the names of section headings in EHR may vary, they still follow certain rules established by usage. The orthographic features were developed to capture subtle writing styles. Each orthographic feature was implemented using regular expressions to capture writing rules of section headings in terms of spelling, hyphenation, and capitalization as shown in Table 3. If the current word matched the defined orthographic feature, its feature value was 1. Otherwise, the value was 0.

### 2.7. Lexicon Features

This work developed four lexicon features. One was the binary lexicon matching feature, whose value was 1 if the current word was a substring of the terms in a lexicon. The strings of the “str” column in the SecTag section header terminology (download from http://knowledgemap.mc.vanderbilt.edu/research/content/sectag-tagging-clinical-note-section-headers) were collected to compile a dictionary for the lexicon feature. Furthermore, in the SecTag terminology, section headings were defined within a hierarchy and were associated with a level of information to indicate their location within a tree. Each heading string was also normalized to a unique string. Both the associated level information and normalized section strings were also encoded as additional lexicon features.

Finally, all section-heading strings collected were tokenized to generate tokens, and their occurrences within the source section string were recorded. After examining all of the section headings, the occurrence information for each token was represented using the values shown in Table 4. The encoded information became the lexicon occurrence feature, and the represented feature value corresponding to the current input token was used as the feature value.

### 2.8. Semantic Features

Some abbreviated section-heading strings are ambiguous. For example, the heading “CC” can either refer to “chief complaint” or “carbon copy” in a clinical narrative. This work developed a binary feature to check whether the current line matches the following pattern “M∖s?∖.?∖s*∗*D∖s?∖.?∣Dr∖s?∖.?∣PCP” to resolve the ambiguity.

### 2.9. Layout Features

Given the variety of layouts of EHRs, the original line breaks of the raw text can guide a supervised learning method to determine the section headings that lead section blocks. This work developed layout features to capture the line break information. In our implementation, for a given split sentence, if its previous line in the original raw text was an empty line, the value of the layout feature was 1, or otherwise it was 0. Take the line starting with the section “Name:” of Table 1 as an example. The value of the layout feature with block size 1 would be 0, but the value of the “CC:” line was 1. The block size for the layout features was set to six, meaning that, for a given sentence, the preceding and the following three lines were considered.

## 3. Experiment

### 3.1. Experiment Configurations

This work follows the i2b2 2014 shared task to divide the compiled section recognition heading corpus into three subsets: set 1 (521 records), set 2 (269 records), and a testing set (514 records). In our experiments, set 1 data was used as the training set for the linear-chain CRF model, and set 2 data was used to develop features. Finally, the testing set was used to assess the performance of the developed model trained on the merged dataset (set 1 + set 2).

The standard precision, recall, and *F*-measure (PRF) metrics were used to evaluate the performance of the developed CRF model and its comparison with dictionary-based methods. Precision and recall were defined as follows:

(2) Precision=the number of correctly recognized section-heading chunksthe number of recognized section-heading chunks,Recall=the number of correctly recognized section-heading chunksthe number of true section-heading chunks,F-measure=2×P×RP+R.

This work defined a correctly recognized section-heading chunk (a true positive case) as a case in which the text span of the recognized section heading completely matches that of the manually annotated heading. Therefore, a false positive (FP) case included any unmatched section headings generated by the computer.

### 3.2. Dictionary-Based and Sentence-Based Section Recognition Methods

To serve as comparisons to our CRF-based model, this work developed two dictionary-based methods based on the maximum matching algorithm as baseline systems. The only difference between the two dictionary-based methods is that the second method filters out all matched section-heading candidates that were not at the beginning of a paragraph.

Three dictionaries were used by the dictionary-based methods: the SecTag section header terminology (the “SecTag” configuration), the section-heading names collected from a training set (the “Training” configuration), and the union of the two dictionaries (the “SecTag + Training” configuration). This work also generated variations for all of the terms in the three dictionaries to improve the coverage of section-heading strings. For example, if the section heading “DISCHARGE MEDICATIONS” exists, the following heading strings were also generated for matching: “Discharge Medications”, “Discharge medications”, “discharge medications”, and their base form “discharge medication”. Stop words such as “and” and “of” were also removed. Finally, all generated strings were further attached with a colon or a dot, such as “discharge medications:” and these variations are also included for matching.

Moreover, to further evaluate the proposed token-based formulation, a sentence-by-sentence classification method based on the maximum entropy (ME) was developed for comparison. The sentence-based segmentation model followed the formulation shown in Table 1 and classified sections in two steps. The model first recognizes the boundary of each section and then applies a regular expression rule to extract the section heading part before the colon from the sentence annotated with the “B” tag. The feature sets used for the one-step approach proposed in [17] were implemented for the method.

### 3.3. Experiment Results

The experiment results of six methods, including the dictionary-based methods, sentence-by-sentence method based on the ME model, and the proposed token-based formulation with the CRF model, were shown in Table 5. The best recall on both datasets was achieved by the dictionary-based method 2 with the section names from the training set and SecTag. By using the section-heading strings from SecTag, the recall of the dictionary-based method can be improved, while the precision is decreased. In comparison to the dictionary-based method 1, method 2 achieved better precision regardless of the dictionaries used. The sentence-based formulation achieved better precision in comparison with the dictionary-based methods.

On the other hand, the token-based formulation noticeably outperformed both the dictionary-based and the sentence-based methods in terms of the *P*- and *F*-scores. On the test dataset, the token-configuration achieved a *P*-score of 0.96, which outperformed the best configuration of the dictionary-based method and the sentence-based formulation by 0.197 and 0.106, respectively. In addition, the token-based formulation based on CRF model achieved the best *F*-score of 0.942. Similar trends can also be observed on the development set.

## 4. Discussion

### 4.1. Error Analysis

As shown in the previous section, the token-based formulation method obviously outperformed the dictionary-based approach and the sentence-by-sentence formulation. The diversity of section-heading names of EHRs is the main factor that resulted in the large performance gap between the machine learning- and dictionary-based methods. A physician can combine any section-heading names in an EHR to form a new section or insert any supplemental information in a section heading. For example, the bold texts in the two section headings “Meds (**confirmed with patient**)” and “DATA (**08/25/61**):” are supporting information. These cases cannot be handled by the dictionary-based method, since dictionaries are unable to cover all variations of such.

The sentence-based formulation could somewhat resolve this issue, but it relied on additional heuristics rules or patterns to separate the content from its section heading. Unfortunately, the possible mentions of section headings vary among EHRs, and heuristics rules may not be competent in distilling the section heading, which further leads to the loss of important information. For instance, in our dataset, the sentence-based formulation cannot determine the section “Assessment and plan” from the description “Assessment and plan Cardiomyopathy. Continue present medications. …” since the section-heading mention did not contain an explicit end boundary. By contrast, the token-based formulation based on the CRF model is capable of identifying these section headings in one step, because the sequential labeling formulation can model the dependency between tokens.

In addition, the results showed that the inclusion of terminology from SecTag led to a decrease in precision. This was caused by the various section headings of different granular levels within the SecTag content. For example, it included terms like “toenail exam” and “muscle tone exam”, which usually does not belong to the topmost section headings.

Through error analysis, the error cases of the developed token-based formulation were divided into two categories: false negative (FN) and false positive (FP) cases. Some errors turned out to increase the number of both FN and FP cases. These errors were propagated from the tokenization errors that may result from transcription errors or erroneous digitalization of EHRs. Take the following tokenized paragraph as an example:

“… renal cell** ca*Family History*** Family history is positive for diabetes … as a floor covering installer. ***Habits*** The patient is a** smoker**.** The** patient's alcohol intake may be excessive. ***Review of Systems*** *ROS*: quite nauseated now and unable to give** details*Medications***lipitor 40”

The texts highlighted in italic and bold denote the annotated section headings and the tokenization errors, respectively. Since our formulation for the section-heading recognition task is based on the sequential labelling of tokens, our CRF model will generate section-heading strings like “caFamily History”, “.Habbit”, “.Reviewof Systems ROS:”, and “detailsMedications”. Each of these heading strings corresponds to one FP and one FN case. The FP and FN cases are discussed individually in the following subsections.

### 4.2. FN Error Cases

The test set contained some topmost section headings that are rarely used in EHRs. These headings, such as “microbiology” and “habits”, only appeared a few times in the training set. Due to the sparseness of these section names, it was difficult for the machine learning-based section tagger to recognize these instances. We also observed that some records adopted nonstandard or idiosyncratic topmost section headings along with abbreviations, which made it difficult to recognize them. Some nonstandard section headings or abbreviations found in the test set included “All” for “allergy” and “ROS” for “Review of Systems”.

### 4.3. FP Error Cases

Occasionally, the trained CRF-based section tagger recognized nonsection parts or probable subsection headings of an EHR, which became the main source of FP cases. For instance, in the following snippet of a record, “The patient is a 75-year-old white female with past medical history significant for throat cancer”, the tagger erroneously identified the nonsection description “medical history” as a section heading. In addition, some section headings such as “laboratory” can be the topmost section headings in one EHR but are not the topmost headings in others. This may also have contributed to the occurrence of FP/FN cases.

### 4.4. Effect of the Layout Features

To study the effect of the proposed layout features, this work trained an additional CRF model excluding the layout features and compared its performance to the model with all proposed feature sets. The comparison is shown in Table 6.

With the layout feature, both the precision and the recall of the CRF-based method were improved on the development and test datasets. The results indicated that adding the layout feature enabled the CRF model to recognize section headings that did not appear in the training set. For example, section headings such as “HCP/FAMILY CONTACT”, “INDICATIONS FOR TPN”, “Allergies or adverse reactions”, “Course on floor”, and “Oncology CONSULTATION NOTE” were not present in the training set. Nevertheless, their clear layout enabled the model with layout features to recognize them accurately.

This work further simulates the scenario when the EHR data did not contain explicit layout format by removing all the original empty line breaks among all text chunks. After removing the layout information, the developed CRF model with all proposed features was again applied on the data. As shown in Table 7, the developed CRF model still achieves satisfying *F*-scores on both datasets and outperforms the *F*-score of the best dictionary-based method by at least 0.0615. The results also reveal that, without the layout information, our CRF model can achieve a better precision with a decreased recall.

### 4.5. Comparison with Other Section-Heading Recognition Methods

Much work has been done on the segmentation of texts in the general or biomedical domains [18–22]. The rationale behind these works is the assumption that there exists a boundary between a sentence and the next. Statistical models are then constructed to assign a probability to the start or the end of every sentence that appeared in a section of an unstructured text.

By contrast, studies and resources related to the recognition of EHR sections are still very limited. Ganesan and Subotin [12] proposed L1-regularized logistic regression model that is capable of recognizing the header, footer, and all of the top-level sections of a clinical note. Tepper et al. [17] showed that the two-step approach which first recognized the section headings followed by their categorization achieved a better performance than the one that combines the two tasks in one step. Li et al. [11] proposed using a hidden Markov model to recognize section headings and normalize them to 15 possible section types. Denny et al. [23] proposed the SecTag algorithm, which used the SecTag terminology to recognize all candidate section headers, and then calculated the Bayesian probability for each candidate in a given text segment. Candidates with low scores were discarded afterwards.

All of the previous works stated formulated the section-heading recognition problem as a classification task that classifies each line of a document or an EHR to a certain category, which is generally referred to as document zoning or sentence classification. For instance, the second column of Table 1 shows the corresponding category for each line in the first column based on the document zoning formulation. Our sentence-based formulation based on ME resembled the traditional approach. Apparently, this formulation requires an additional effort to distinguish the section heading from other contexts. For instance, after recognizing the line “ALL: NKDA, Intolerance to Inderal” as the beginning of a section segmentation, additional postprocessing is required to distinguish the section-heading string “ALL:”. On the other hand, this approach assumed that the section-heading strings themselves can be recognized by using simple heuristics rules or patterns. Our sentence-based method followed the idea and developed an additional regular expression pattern to extract section-heading parts from the first line of a recognized section boundary. Consistent with the highly implicit section-heading boundary distribution observed on the corpus (55.58%), if the heuristics patterns were removed from our sentence-based formulation configuration, the precision and recall drop significantly and resulted in an *F*-score of 0.4062 on the test set. By contrast, our token-based formulation presented an integrated solution without the requirement to develop additional heuristics rules for isolating the heading from the surrounding section contents.

## 5. Conclusions

Due to the lack of openly available section recognition tools and corpora, most works still used the dictionary-based approaches with section-specific lexicon to recognize section headings in practice. This work presents the first attempt to formulate the section-heading recognition problem as a token-based sequential labeling task and employed CRF model with a set of features developed to recognize section headings in EHRs. Compared with the traditional sentence classification formulation, the proposed token-based formulation proposed an integrated solution without the requirement to develop additional heuristics rules for isolating the heading from the surrounding section contents. Our formulation based on the CRF model was compared with the traditional formulation and two dictionary-based methods with expanded section-heading terms. The experiment results showed that the proposed token-based formulation evidently outperformed the sentence-based formulation and the dictionary-based approaches in terms of precision and *F*-scores. The proposed layout features, which captured the line break information, can model the original layout given by medical doctors with the intention of increasing readability. Implementing the layout features into our method resulted in an improved recall of section-heading recognition, which was supported by the experiment results.

Nevertheless, the current work remains limited in certain perspectives, as the developed corpus and the proposed method only examine the topmost sections. Subsections which may also contain important and distinct information were not taken into consideration. In the future, the research attempt is to extend the corpus to include the subsection heading annotations and section-heading normalization information. For instance, section-heading strings like “presenting complaints”, “presenting problems”, and “reason for encounter” are all normalized to “chief complaints”. This investigation may help enhance the meaningful use of EHRs to facilitate and improve the quality of health care.

## Figures and Tables

**Table 1:** A sample of discharge summary.

**Record date:** 2088-07-03	**B**
**Name:** Younger, T Eugene	**B**
**Date of Admission:** 7/2/88	**B**
	**I**
**CC:** Lightheadedness, vertigo, and presyncopalsx × several episodes	**B**
	**I**
**HPI:.** 64 yoM w/significant PMH for CAD, HTN, GERD, and past cerebral embolism presents w/6 hour history of vertiginous symptoms, dizziness, lightheadedness, and feeling “like [he] was going to pass out”. The pt recalls…	**B**
	**I**
**ROS:** +tinnitus, −weakness/change in sensation, ataxia, aura-like sx, post-episode confusion	**B**
	**I**
**PMH:**	**B**
**CAD:** 2075 PTCA w/Angioplasty to LAD, Stress (3/88): rev. anterolateral ischemia, Cath (5/88): 3v disease: RCA 90%, LAD 30% mid, 80% distal, D1 70%, D2 40% and 60%, LCx 30%, OM2 80%	**B**
	**I**
**Meds (Updated 7/20)**	**B**
Atenolol 25/50 mg qAM/qPM	**I**
ASA 325 mg qD	**I**
**ALL:** NKDA, Intolerance to Inderal	**B**
	**I**
**FHx:**	**B**
+HTN: mother/brother	**I**
**SocHx:** Lives by himself separated.	**B**
	**I**
**PE:**	**B**
VS:	**I**
Gen: Well-nourished male, NAD	**I**
HEENT: MMM, OP clear	**I**
Neck: JVP about 9 cm.	**I**
	**I**
**LABS:**	**B**
Sodium 140 135–145 mmol/L 07/02/88 11:21 147(H) 10/08/82 13:24	**I**
Potassium 4.1 3.4–4.8 mmol/L 07/02/88 11:21	**I**
EKG: Sinus brady @ 60, w/LAD, ICVD (QRS 108), NS St/T wave changes.	**I**
CXR: Pending	**I**
	**I**
**Impression:** 64 yo male w/significant CAD, past cerebral emboli, presents w/sx …	**B**
**Plan:**	**B**
(1) Vertigo: Clinically peripheral disease. If central, would not expect to be affected by motion, be able to be eextinguished, and so forth.	**I**
Fall precautions	**I**
R/o cardiac ischemia: Troponins, monitor, and so forth	**I**
Betty Kaitlin Wood, MD	**I**

**Table 2:** Statistics of the section heading recognition corpus. Since the corpus only contained the topmost sections, several different concepts or representations may be included in each section heading category. For instance, “Personal Histories” included the occupation, daily activity amount, substance history, and allergies.

Section	Description	Number	Percentage
Chief Complaints	A statement describing the symptoms, problems, diagnoses, or other factors that are the reason of a medical encounter.	803	5.7%
Present Illness	Separated paragraphs summarizing chief complaints related history.	843	6.0%
Personal Histories	A merged concept of individual related histories, including past medical history, past surgical history, social history, and allergy.	2701	19%
Family Histories	The health status of parents, children, siblings, and spouse, whether dead or alive.	486	3.4%
Physical Examinations	The process by which a medical professional investigates the body of a patient for signs of disease.	1104	7.9%
Laboratory Examinations	Biochemical studies performed in clinical laboratory.	401	2.8%
Radiology Reports	Image studies. Some examples are X-ray, CT, MRI, and PET.	87	<1.0%
Data	A merged concept including laboratory examinations and radiology reports.	103	<1.0%
Impression	Medical diagnoses judged by doctors, also called assessments.	884	6.3%
Recommendations	Treatments toward impressions, also called plans.	468	3.3%
Others	Other section headings not included in the categories above, for example, patient ID, doctor ID, and hospital ID.	6081	43.6%

Total		13,962	100%

**Table 3:** Orthographic features.

Feature name	Regular expression
ALLCAPS	^∧^[*A* − *Z*] + $
CAPSMIX	^∧^[*A* − *z*] *∗* ([*A* − *Z*] [*a* − *z*]∣[*a* − *z*] [*A* − *Z*]) [*A* − *z*] *∗* $
INITCAP	^∧^[*A* − *Z*]
PUNCTUATION	^∧^[∖.:]$

**Table 4:** Occurrence information.

Description	Feature value
The token was not matched.	000
The token only appeared in the first token among all section headings.	001
The token only appeared in the middle token among all section headings.	010
The token only appeared in the last token among all section headings.	100
The token appeared in both the first and middle tokens among all section headings.	011
The token appeared in both the middle and last tokens among all section headings.	110
The token appeared in both the first and last tokens among all section headings.	101
The token appeared in all places among all section headings.	111

**Table 5:** Performance comparison among different methods.

Dataset	Configuration	*P* (%)	*R* (%)	*F* (%)
Set 2	Dict. method 1 (SecTag)	19.9	79.31	31.82
	Dict. method 1 (set 1)	52.18	94.04	67.12
	Dict. method 1 (SecTag + set 1)	23.19	**94.99**	33.47
	Dict. method 2 (SecTag)	41.19	79.31	54.22
	Dict. method 2 (set 1)	**75.5**	94.04	**83.76**
	Dict. method 2 (SecTag + set 1)	45.33	**94.99**	61.37
	Sentence-based formulation (ME)	81.54	82.16	81.85
	Token-based formulation (CRF)	**95.48**	92.66	**94.05**

Test	Dict. method 1 (SecTag)	21.15	80.23	33.47
	Dict. method 1 (set 1 + set 2)	54.13	94.87	68.93
	Dict. method 1 (SecTag + set 1 + set 2)	24.38	**95.48**	38.84
	Dict. method 2 (SecTag)	41.72	80.23	54.89
	Dict. method 2 (set 1 + set 2)	**76.37**	94.84	**84.6**
	Dict. method 2 (SecTag + set 1 + set 2)	45.59	**95.48**	61.71
	Sentence-based formulation (ME)	85.46	85.54	85.5
	Token-based formulation (CRF)	**96.04**	92.4	**94.19**

**Table 6:** Performance comparison for the layout features.

Dataset	Configuration	*P* (%)	*R* (%)	*F* (%)
Set 2	CRF-based without layout features	94.8	90.72	92.72
	CRF-based with layout features	**95.48**	**92.66**	**94.05**

Test	CRF-based without layout features	95.13	90.5	92.76
	CRF-based with layout features	**96.04**	**92.4**	**94.19**

**Table 7:** Performance for EHR data without layout information.

Dataset	*P* (%)	*R* (%)	*F* (%)
Set 2	97.2	84.88	90.62

Test	97.59	84.81	90.75
